# Substance P and NK‐1R in Dengue: A First Serological Investigation

**DOI:** 10.1002/hsr2.72820

**Published:** 2026-07-27

**Authors:** Riffat Mehboob, Imran Shahid, Shahzad Ahmad, Noreen Sarwar, Akash John, Zeeshan Mehboob, Majid Almansouri, Waleed Badoghaish

**Affiliations:** ^1^ Rotogen Biotech Bethesda Maryland USA; ^2^ Lahore Medical Research Center Lahore Pakistan; ^3^ Department of Pharmacology and Toxicology, Faculty of Medicine Umm Al‐Qura University Makkah Saudi Arabia; ^4^ Faculty of Medicine and Health Sciences The University of Buckingham Buckingham UK; ^5^ Institute of Microbiology University of Veterinary and Animal Sciences Lahore Pakistan; ^6^ Department of Clinical Biochemistry, Faculty of Medicine King Abdulaziz University Jeddah Saudi Arabia; ^7^ Department of Internal Medicine, Faculty of Medicine University of Tabuk Tabuk Saudi Arabia

**Keywords:** COVID‐19, dengue infection, inflammation, neurokinin 1 receptor, respiratory infections, substance P

## Abstract

**Background:**

Dengue fever lacks reliable biomarkers to explain its complex pathophysiology. Substance P (SP) and its receptor, the Neurokinin‐1 receptor (NK1R), known regulators of inflammation and vascular permeability in viral infections, may play a role in dengue but remain unexplored.

**Aim/Objective:**

To investigate the levels of SP and NK1R in serum samples of diagnosed dengue patients.

**Methods:**

Participants were selected from a pool of diagnosed dengue fever patients using a convenience sampling technique (*n* = 40), along with a control group of healthy individuals (*n* = 12). Serum samples were collected, and SP and NK1R levels were measured using enzyme‐linked immunosorbent assay (ELISA). Complete blood count (CBC) parameters were also evaluated.

**Results:**

The mean NK1R levels in the control and case groups were 2.681 ± 3.147 and 0.57 ± 0.131, respectively. NK1R levels were significantly lower in dengue patients compared with controls (*p* < 0.0001). SP levels did not differ significantly between male and female dengue patients (Mann–Whitney *U* = 85, *p* = 0.899).

**Conclusion:**

NK1R may be associated with dengue pathogenesis through its potential involvement in vascular permeability, immune activation, and cytokine responses. These findings suggest a possible connection between NK1R and the pathophysiological mechanisms underlying dengue infection.

## 1 Introduction

Dengue is the most common arboviral illness worldwide, an acute arthropod‐borne viral infection that heavily impacts many tropical and subtropical countries in terms of both socioeconomic load and disease burden [1]. Dengue is spreading faster than any other infectious illness, according to the Global Burden of Disease survey, with a 400% rise in only 13 years (2000–2013) [2]. Despite the fact that dengue is still considered a neglected tropical illness, during the last 10 years, there has been an exponential growth in funding for the development of vaccines and innovative vector control strategies [3]. Dengue, which is mostly prevalent in tropical and subtropical regions and is spread by *Aedes mosquitoes*, is home to over 3 billion people [4]. Dengue infections were thought to affect 400 million people annually, of whom 25% had clinical symptoms and caused 1 million disability‐adjusted life years (DALYs) worldwide [5]. Although the mortality rate from severe dengue is relatively low compared with other infectious diseases, it still causes an estimated 20,000 deaths annually worldwide, imposing a substantial health and economic burden in endemic regions [6, 7]. Seventy‐five percent of cases of dengue are said to occur in Asia, with Latin America and Africa following [1].

Vector salivary peptides provide indirect yet compelling evidence for the involvement of substance P (SP) pathways in dengue infection. Specifically, sialokinins I and II, tachykinins isolated from *Aedes aegypti saliva*, exhibit structural similarities to mammalian SP. These peptides activate similar signaling pathways, suggesting an evolutionary conservation of tachykinin–NK1R signaling. This conservation may play a significant role in modulating the pathophysiology of dengue by affecting vascular permeability and immune responses [8, 9, 10].

SP, a neuropeptide, exerts its proinflammatory and vasoregulatory effects predominantly through the neurokinin‐1 receptor (NK1R), a G‐protein‐coupled receptor expressed on endothelial and immune cells. This receptor‐mediated signaling cascade is critical in the inflammatory response, which is central to the vascular leakage and immune activation observed in dengue pathogenesis. Using the antagonist aprepitant, the researchers investigated how inhibiting the NK1R affected the function of sialokinin. Because sialokinin's sequence resembles that of tachykinins found in mammals, it is classified as a tachykinin. For contrast, SP, an endogenous NK1R agonist, was used. The investigations validated that the longer probing duration and decreased blood‐feeding efficiency seen in sialokinin‐deficient mosquitoes (sialokinin‐KO mosquitoes) were caused by sialokinin's interaction with the vertebrate tachykinin receptor, particularly NK1R. The blood vessel endothelium contained NK1R. The investigation assessed how sialokinin affected NK1R in the endothelium and showed that it was responsible for the biological reactions that were seen [11, 12].

Further research was necessary since the mechanism of action of these cytokine‐like compounds in the pathophysiology of dengue remained unclear. Although the mortality rate from severe dengue is low, endemic situations can place a significant financial and resource strain on health care. Regrettably, despite significant advancements in our knowledge of disease etiology and substantial investment in the search for antiviral drugs, the development of effective therapies has unfortunately proceeded slowly. This work explores the relationship between dengue fever and the vasodilatory effects of SP and its receptor, NK1R.

## 2 Methods

This cross‐sectional study was conducted at Lahore Medical Research Center between July 2024 and October 2024. Participants were selected using a convenience sampling technique and included 40 diagnosed dengue fever patients along with a control group of 12 healthy individuals without dengue infection. Ethical approval was obtained from Lahore Medical Research Center, Lahore, Pakistan, under reference number LMRC/001/2024. Diagnosis of dengue was confirmed through (NS1 antigen/IgM ELISA/RT‐PCR) following WHO guidelines. Inclusion criteria were patients with confirmed dengue infection who provided written informed consent, while exclusion criteria included patients with co‐infections, chronic liver disease, immunosuppressive therapy, or pregnancy. Healthy controls were selected from hospital staff/volunteers/blood donors with no history of recent febrile illness. A convenience sample of 40 dengue patients and 12 healthy controls was included in the study, which provided an estimated statistical power of approximately 80% with *α* = 0.05. Each participant gave informed consent before the collection of demographics, medical, and clinical data. Venous blood was drawn under aseptic conditions, and serum was extracted by centrifugation at 4500 rpm for 5 min and stored at the appropriate temperature until further analysis. Complete blood count (CBC) parameters, including hemoglobin, MCH, MCHC, RDW‐SD, lymphocytes, neutrophils, and monocytes, were analyzed in all dengue patients using standard hematological procedures to determine possible gender‐based variations. Serum levels of SP and NK1R were measured using commercially available enzyme‐linked immunosorbent assay (ELISA) kits (BT Lab Human Substance P ELISA kit, Cat. No. E1528Hu; BT Lab Human NK1R ELISA kit, Cat. No. E6938Hu) according to the manufacturer's instructions. Statistical analysis was carried out using GraphPad Prism version 8.0.2 (GraphPad Software, San Diego, CA, USA). The distribution of data for SP, NK1R, and all CBC parameters was assessed for normality using the Shapiro–Wilk test. Based on this assessment, descriptive statistics for normally distributed data are presented as mean ± standard deviation (SD), and non‐normally distributed data are presented as median and interquartile range (IQR). For the primary, pre‐specified analyses (case vs. control), comparisons between two independent groups were conducted using two‐sided unpaired Student's *t*‐test for normally distributed data, and two‐sided Mann–Whitney *U* test for non‐normally distributed data. Exploratory subgroup analyses (e.g., male vs. female) employed the same two‐sided tests. For comparisons across more than two groups (e.g., age groups), two‐sided one‐way ANOVA was used for normally distributed data, and two‐sided Kruskal–Wallis test was used for non‐normally distributed data. An a priori significance level of *α* = 0.05 was established for all statistical tests. Confidence intervals (95% CI) for the difference between medians/means are reported for key comparisons. This analytical approach follows the SAMPL (Statistical Analyses and Methods in the Published Literature) [13].

## 3 Results

A total of 40 dengue patients were included in this study, comprising 34 males (85.0%) and 6 females (15.0%), with mean ages of 30.0 ± 13.62 years and 31.7 ± 5.16 years, respectively. CBC analysis showed no significant gender‐based differences in most parameters except hemoglobin, MCH, MCHC, RDW‐SD, lymphocytes, neutrophils, and monocytes. The respective *p*‐values were 0.0007, < 0.0001, 0.021, 0.0036, 0.0296, 0.0176, and 0.0005 (Table 1).

**Table 1:** Demographic and hematological parameters of dengue patients.

Variables	Male (*n *= 34)	Female (*n* = 6)	Total(*n* = 40)	*p* value
Frequency (%)	34 (85.0%)	6 (15.0%)	40 (100.0%)	
Age (years)	30 ± 13.62	31.67 ± 5.164	30.25 ± 12.68	0.7704
WBC (10^9^/L)	4.266 ± 2.366	3.800 ± 1.431	4.197 ± 2.243	0.6449
RBC (10^6^/ml)	5.002 ± 0.4148	4.990 ± 0.1885	5.000 ± 0.3875	0.9453
Hemoglobin (g/dL)	13.37 ± 0.8133	11.87 ± 1.415	13.15 ± 1.055	0.0007**
MCV (fL)	72.17 ± 18.60	72.00 ± 2.204	72.15 ± 17.13	0.9825
PCV(HCT)	52.28 ± 19.76	37.37 ± 4.528	50.04 ± 19.03	0.0764
MCH (Pg)	26.78 ± 1.924	22.80 ± 0.6753	26.18 ± 2.293	< 0.0001****
MCHC (%)	32.28 ± 0.5478	31.73 ± 0.2066	32.20 ± 0.5458	0.021*
RDW‐CV (%)	22.14 ± 29.19	14.80 ± 0.5586	21.04 ± 26.98	0.5459
RDW‐SD (%)	48.89 ± 5.338	42.00 ± 1.713	47.86 ± 5.540	0.0036**
Platelets (10^3^/ml)	104.7 ± 26.40	124.0 ± 21.69	107.6 ± 26.43	0.0997
MPV (fL)	8.782 ± 1.406	9.200 ± 1.342	8.845 ± 1.388	0.5035
PDW (%)	12.58 ± 2.759	12.57 ± 1.792	12.58 ± 2.617	0.9878
PCT (ng/ml)	0.086 ± 0.0278	0.11 ± 0.036	0.089 ± 0.029	0.0695
P‐LCR (%)	23.75 ± 9.937	23.97 ± 10.66	23.79 ± 9.905	0.9608
Lymphocytes (%)	29.29 ± 11.97	40.67 ± 6.088	31.00 ± 11.96	0.0296*
Neutrophils (%)	62.71 ± 13.10	49.00 ± 7.099	60.65 ± 13.28	0.0176*
Monocytes (%)	5.000 ± 1.875	8.000 ± 0.8944	5.450 ± 2.062	0.0005***
Eosinophils (%)	2.41 ± 0.857	2.33 ± 0.516	2.4 ± 0.81	0.9344

*Note:* Values are expressed as mean ± SD.

Abbreviations: Hb, hemoglobin; MPV, mean platelet volume; MCH, mean corpuscular hemoglobin; MCHC, mean corpuscular hemoglobin concentration; MCV, mean corpuscular volume; PCV, packed cell volume; PLT, platelets; PDW, platelet distribution width; PCT, plateletcrit; P‐LCR, platelet large cell ratio; RBC, red blood cells; RDW‐CV, red cell distribution width–coefficient of variation; RDW‐SD, red cell distribution width–standard deviation; WBC, white blood cells.

* *p* < 0.05

** *p* < 0.01

*** *p* < 0.001

**** *p* < 0.0001.

Serum levels of SP and NK1R were measured in dengue patients (cases) and healthy controls (Table 2). SP levels were 355.7 ± 576.3 pg/mL in cases and 615.0 ± 177.2 pg/mL in controls, with no statistically significant difference (*p* = 0.1246, Mann–Whitney *U* test). NK1R levels were 0.57 ± 0.131 ng/mL in cases and 2.681 ± 3.147 ng/mL in controls, showing a significant difference between groups (*p* < 0.0001, unpaired *t*‐test) (Table 2).

**Table 2:** Serum Substance P (SP) and NK1R levels in dengue patients (cases) and healthy controls.

Markers	Case	Control	*p* value
SP (pg/mL)	355.7 ± 576.3	615.0 ± 177.2	0.1246
NK1R (ng/mL)	0.57 ± 0.131	2.681 ± 3.147	< 0.0001****

*Note:* Values are expressed as mean ± SD.

Abbreviations: NK1R, neurokinin‐1 receptor; SP, substance P.

**** *p* < 0.0001.

Within the dengue patient group, no significant differences were observed in serum SP or NK1R levels between males and females. SP levels were 161.0 pg/mL (IQR: 85.3–372.5, *n* = 34) in males and 179.0 pg/mL (IQR: 156.8–211.5, *n* = 6) in females (Mann–Whitney *U* = 85, *p* = 0.899). NK1R levels were 0.57 ± 0.13 ng/mL in males and 0.64 ± 0.14 ng/mL in females (unpaired *t*‐test, *p* = 0.158) (Table 3).

**Table 3:** Comparison of serum substance P (SP) and NK1R levels between male and female dengue patients.

Markers	Male (*n* = 34)	Female (*n* = 6)	Total (n = 40)	*p* value
SP	386.0 ± 620.4	184.0 ± 95.25	355.7 ± 576.3	0.4358
NK1R	0.54 ± 0.121	0.721 ± 0.069	0.57 ± 0.131	0.1578

*Note:* Values are expressed as mean ± SD.

Abbreviations: NK1R, neurokinin‐1 receptor; SP, substance P.

Serum SP and NK1R levels were analyzed across different age groups within the dengue patient cohort (Table 4). SP levels, which were non‐normally distributed, showed variation among age groups, with the highest median in the 31–40‐year group; however, the difference was not statistically significant (*p* = 0.0963, Kruskal–Wallis test). NK1R levels, which were normally distributed, also differed significantly across age groups, with the highest mean observed in the 51–60‐year group (one‐way ANOVA, *p* = 0.0014) (Table 4).

**Table 4:** Distribution of SP and NK1R levels with respect to age groups.

Age groups (yrs)	Frequency	SP (pg/mL)	NK1R (ng/mL)
0–20	8 (20.0%)	101.3 ± 109.5	0.4630 ± 0.142
21–30	16 (40.0%)	230.8 ± 162.7	0.6086 ± 0.073
31–40	8 (20.0%)	787.1 ± 116.1	0.6310 ± 0.155
41–50	6 (15.0%)	530.4 ± 311.7	0.4703 ± 0.056
51–60	2 (5.0%)	124.054 ± 60.84	0.743 ± 0.095
Total	40 (100.0%)	355.7 ± 576.3	0.57 ± 0.131
*p*‐value		0.0963	0.0014**

*Note:* Values are expressed as mean ± SD.

Abbreviations: NK1R, neurokinin‐1 receptor; SP, substance P.

** *p* < 0.01.

**Figure 1:**
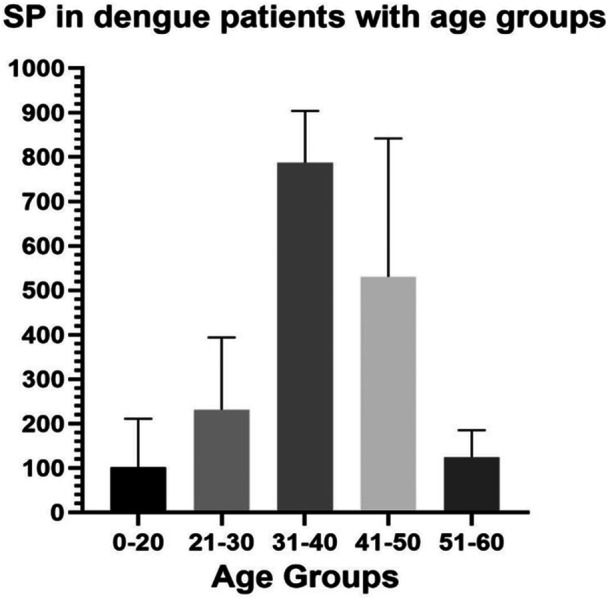
Median serum substance P (SP, pg/mL) levels in dengue patients by age group.

Figure 1 shows that SP levels varied across age groups, with the highest median level observed in the 31–40‐year age group. However, the differences between age groups were not statistically significant (*p* = 0.0963).

**Figure 2:**
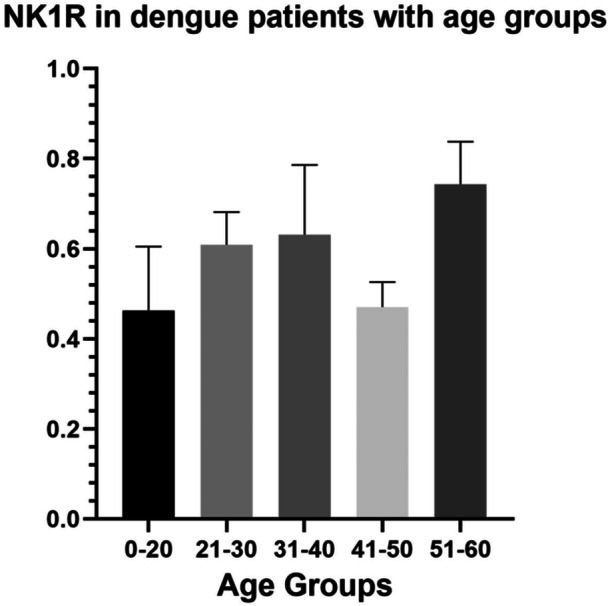
Mean serum Neurokinin‐1 receptor (NK1R, ng/mL) levels in dengue patients by age group.

Figure 2 shows that NK1R levels varied across age groups, with the highest mean level observed in the 51–60‐year age group and the lowest in the 0–20‐year age group. The differences between age groups were statistically significant (*p* = 0.0014).

## 4 Discussion

The study included a thorough examination of demographic features, CBC parameters, and levels of SP and NK1R in dengue patients. This is among the first studies to investigate SP and NK1R levels in dengue patients. The observed variations in CBC parameters between males and females, notably in hemoglobin, MCH, MCHC, RDW‐SD, lymphocytes, neutrophils, and monocytes, may indicate gender‐specific immunological responses to dengue infection. However, additional variables such as genetic predisposition, lifestyle, and comorbidities must be considered [14, 15, 16, 17]. The study examined the relationship between SP/NK1R and dengue infection. While SP levels did not change significantly between the control and case groups, NK1R levels did. This suggests that NK1R may be associated with the pathophysiology of dengue infection. These results are consistent with growing evidence that neuroimmune and endothelial interactions may contribute to dengue pathogenesis [18, 19]. Nonetheless, further research is needed to elucidate the specific mechanisms underlying the link between NK1R and dengue. When comparing these findings to previous research, it is critical to recognize differences in study populations, methodologies, and geographic regions. Some studies have shown gender variations in dengue severity and immunological responses, which support our results. However, the exact CBC parameters found to be significantly different across genders in this study may vary from those reported in other regions [14, 15, 16, 17]. The findings relating to SP and NK1R are notable and provide preliminary insights. Although there was no statistically significant difference in SP levels between the control and case groups, there was a significant difference in NK1R levels, which aligns with the limited literature on dengue neuroimmune components [19, 20]. Future studies should investigate whether similar associations can be generalized to other populations.

The age‐related analysis found significant changes in NK1R levels across age groups, indicating a potential age‐dependent association in dengue infection. In contrast, SP levels did not differ significantly across age groups. These results emphasize the importance of considering age when studying dengue infection [15, 21]. Although the sample size was small, the findings point to a novel direction not previously explored. SP and its receptor, NK1R, are key mediators of neurogenic inflammation and vascular permeability and have been implicated in several viral infections, particularly respiratory diseases such as influenza and SARS‐CoV‐2, where NK1R antagonists have been proposed as therapeutic agents [22, 23, 24, 25, 26]. In contrast, our study observed significantly lower serum NK1R levels in dengue patients compared with controls, while SP levels did not differ. This divergence from previous viral studies may reflect differences in disease phenotype, timing of sampling, or methodological factors, as most assays detect soluble NK1R fragments rather than membrane‐bound receptors. Additionally, vector‐derived tachykinins, such as sialokinins, could influence host NK1R regulation in dengue. These findings highlight the need for longitudinal and mechanistic studies, including tissue‐level assays, to clarify whether reduced serum NK1R represents receptor downregulation, altered shedding, or assay limitations [27, 28]. Our findings suggest that serum SP/NK1R patterns in dengue may differ from those reported in some respiratory viral infections [22, 24, 25, 26]. This interpretation is consistent with our previous research, including our collaborative clinical trial on the therapeutic potential of aprepitant and dexamethasone combination in severe to critical COVID‐19 patients [24], the identification of NK1R as a promising drug target for COVID‐19 treatment, and our exploration of endothelial cells and angiotensin‐converting enzyme‐II in COVID‐19‐related brain damage [29]. Additional investigations with Peter Oehme into SP in the respiratory tract and neurological manifestations following COVID‐19 infection [25], as well as its role in corona infections and associated brain damage [26], further support the contention that the SP/NK1R pathway may be involved in respiratory viral infections. This may indicate a different SP/NK1R profile in dengue compared with respiratory viral conditions.

The observed association between NK1R levels and dengue infection could be explained by its possible role in modulating vascular and immune responses. Activation of NK1R by SP has been associated with increased endothelial permeability and inflammatory cell recruitment, which are central to dengue pathogenesis. NK1R signaling may also amplify the release of proinflammatory cytokines, potentially contributing to the cytokine storm observed in severe cases. Additionally, neuroimmune interactions involving mast cells and peripheral nerves may further exacerbate vascular leakage and systemic inflammation. These mechanisms suggest that altered NK1R expression may be relevant to disease severity in dengue [20, 21, 27].

Although systemic SP levels were not significantly altered in dengue patients, SP may still modulate immune and endothelial responses locally, influencing vascular permeability and inflammation. The significant reduction in NK1R may suggest altered NK1R‐related signaling in dengue, potentially through altered receptor‐mediated pathways. These results warrant further investigation into SP/NK1R‐mediated mechanisms at the cellular and tissue level [27].

Recently, a study by Deng et al. reported the inhibition of dengue virus (DENV) infection in vitro using the NK‐1R antagonist fosaprepitant dimeglumine, an FDA‐approved anti‐emetic drug. This study highlights the possible involvement of SP/NK‐1R in the underlying mechanism of dengue viral infection, but further exploration is required as this is only one in vitro study. If supported by further evidence, NK‐1R antagonists may serve as a promising therapeutic target for dengue infection [30].

There are very few studies discussing the role of SP in other viral infections. In one previous study, Mehboob R. proposed the involvement of SP/NK1R in COVID‐19 infection [24]. Munoz, Covenas, and Kramer have proposed that SP/NK‐1R may be involved in viral infections such as HIV/AIDS and that NK‐1R antagonists may be a useful treatment strategy in this regard [31]. In an experimental study conducted on a BL6 mouse model, it was observed that SP was upregulated in the brain during West Nile neuroinvasive disease as compared with mock‐infected animals [28]. Interestingly, SP/NK‐1R levels were observed to be raised in viral infections as reported in the above studies, which is contrary to our current study.

This study is limited by its small sample size, single time‐point measurements, and reliance on serum assays that may not reflect tissue‐level NK1R activity. Potential effects of comorbidities, medications, or other individual factors on SP and NK1R levels were not accounted for. Future studies with larger sample sizes should include subgroup analyses to explore these effects and better understand the role of NK1R in dengue pathogenesis.

## 5 Conclusions

This study provides insight into the neuroimmune, hematological, and demographic aspects of dengue infection. The observed gender differences in CBC parameters, along with the predominance of male patients, highlight potential interactions between host characteristics and immune responses in dengue. The evaluation of NK1R and SP as potential markers of dengue infection yielded preliminary findings. While SP levels did not differ significantly between cases and controls, a significant difference was observed in NK1R levels. These findings suggest a possible association between NK1R and dengue infection and underscore the importance of further investigating neuroimmune interactions in the pathophysiology of dengue.

## Author Contributions

**Riffat Mehboob:** conceptualization, methodology, investigation, validation, writing – review and editing, supervision, resources. **Imran Shahid:** project administration, writing – review and editing, methodology, conceptualization. **Shahzad Ahmad:** Manuscript writing, project supervision, critical review. **Noreen Sarwar:** methodology, writing – original draft, data curation, investigation. **Akash John:** writing – review and editing, conceptualization, methodology, writing – original draft, formal analysis, visualization, project administration. **Zeeshan Mehboob:** software, formal analysis, visualization, writing – review and editing, data curation. **Majid Almansouri:** investigation, writing – review and editing, validation, data curation. **Waleed Badoghaish:** Critical review, manuscript draft, analysis and interpretation.

## Ethics Statement

The study was approved by the Lahore Medical Research Center, Lahore, Pakistan (Ethical Clearance Reference Number: LMRC/001/2024).

## Consent

All participants provided written informed consent prior to participating. Participants also provided consent for publication.

## Conflicts of Interest

The authors declare no conflicts of interest.

## Transparency Statement

The Prof. Dr. Riffat Mehboob affirms that this manuscript is an honest, accurate, and transparent account of the study being reported; that no important aspects of the study have been omitted; and that any discrepancies from the study as planned have been explained.

## Data Availability

The data that support the findings of this study are available on request from the corresponding author. The data are not publicly available due to privacy or ethical restrictions.
