# Correction to “Substance P and NK‐1R in Dengue: A First Serological Investigation”

**DOI:** 10.1002/hsr2.73048

**Published:** 2026-08-11

R. Mehboob, I. Shahid, S. Ahmad, et al., “Substance P and NK‐1R in Dengue: A First Serological Investigation,” Health Science Reports 9 (2026): e72820, https://doi.org/10.1002/hsr2.72820.

The affiliation of the author Waleed Badoghaish was incorrect and has been corrected to Department of Internal Medicine, Faculty of Medicine, University of Tabuk, Tabuk, Saudi Arabia.

The online version of this article has been corrected accordingly.

We apologize for this error.

